# Evaluation of Deposition and Clearance of Asbestos (Detected by SEM-EDS) in Lungs of Deceased Subjects Environmentally and/or Occupationally Exposed in Broni (Pavia, Northern Italy)

**DOI:** 10.3389/fpubh.2021.678040

**Published:** 2021-07-20

**Authors:** Silvia D. Visonà, Silvana Capella, Sofia Bodini, Paola Borrelli, Simona Villani, Eleonora Crespi, Claudio Colosio, Carlo Previderè, Elena Belluso

**Affiliations:** ^1^Unit of Legal Medicine and Forensic Sciences, Department of Public Health, Experimental and Forensic Medicine, University of Pavia, Pavia, Italy; ^2^Department of Earth Sciences, University of Torino, Turin, Italy; ^3^Interdepartmental Center for Studies on Asbestos and other Toxic Particulates “G. Scansetti”, University of Torino, Turin, Italy; ^4^Unit of Biostatistics and Clinical Epidemiology, Department of Public Health, Experimental and Forensic Medicine, Pavia University, Pavia, Italy; ^5^Laboratory of Biostatistics, Department of Medical, Oral and Biotechnological Sciences, University “G. d'Annunzio”, Chieti, Italy; ^6^Occupational Health Unit, Santi Paolo e Carlo Hospital, Milan, Italy; ^7^Department of Health Sciences, University of Milan, Milan, Italy

**Keywords:** asbestos, malignant mesothelioma, SEM-EDS, asbestos clearance, chrysotile, lung fiber burden

## Abstract

Biodurability is one of the main determinants of asbestos hazardousness for human health. Very little is known about the actual persistence of asbestos in lungs and its clearance, nor about differences in this regard between the different mineralogical types of asbestos. The aim of the present study was to evaluate the amount, the dimensional characteristics and the mineralogic kinds of asbestos in lungs (measured using SEM-EDS) of a series of 72 deceased subjects who were certainly exposed to asbestos (mainly crocidolite and chrysotile) during their life. Moreover, we investigated possible correlations between the lung burden of asbestos (in general and considering each asbestos type), as well as their dimension (length, width, and l/w ratio) and the duration of exposure, the latency- in case of malignant mesothelioma (MM), the survival and the time since the end of exposure. In 62.5% of subjects, asbestos burden in lungs was lower that the threshold considered demonstrative for occupational exposure. In 29.1% of cases no asbestos was found. Chrysotile was practically not detected. The mean length of asbestos fibers and the length to width ratio were significantly related to the duration of exposure to asbestos. No other statistically significant correlations were found between the amount and dimensional characteristics of asbestos (nor with the relative amount of each asbestos type) and the other chronological variables considered. In conclusion, it was pointed out that chrysotile can be completely removed from human lungs in <8 years and, instead, amphiboles persist much more time. The present results suggest, as well, that the finding of no asbestos in lungs cannot rule out the attribution of MM to asbestos (in particular, chrysotile) inhaled in an occupational setting. This point is of crucial importance from a legal point of view.

## Introduction

The relation between exposure to asbestos and malignant mesothelioma (MM) is well-documented, but many issues concerning the etiopathogenesis of this disease, as well as of other asbestos-related diseases (ARD), are still debated. Among many others, the relation between the duration of exposure and, on the other hand, the time between the end of exposure and death, on lung fiber burden in subjects died from MM, is still not fully understood.

Such questions are of paramount importance, especially considering the particularly prolonged latency time between asbestos exposure and the development of ARD, suggesting that many biological events take place during the time between exposure and the clinical manifestations of ARD (asbestosis, MM, lung cancer).

Despite the open, intense debate still ongoing about the different hazardousness and cancerogenic potential of the various type of asbestos (1–4); according to the World Health Organization (WHO) and the International Agency for Research on Cancer (IARC), all types of asbestos are classified as class I carcinogens (5). Chrysotile has been demonstrated to be a potent carcinogen in animals (6).

The dynamics of lung clearance of asbestos have been largely investigated on animal models, but few studies on humans address this issue. In 1987 Churg and De Paoli observed, under electron microscope, autoptic lung samples of two groups of asbestos workers whose last exposure occurred, respectively, 2 and 12 years before death; they found that the ratio between chrysotile and tremolite did not show any variation with time, suggesting that chrysotile clearance in lungs occurs shortly after exposure (7). The same authors published two reviews, including also experimental data on animals (8, 9), confirming their previous results. On the basis of lung content analysis (performed using analytical transmission electron microscopy), other authors suggested that the dose of chrysotile required to cause MM is higher than the dose of amphiboles (10). Therefore, they hypothesized that amphiboles are more potent carcinogens compared to chrysotile (in relation with its quicker degradation). Moreover, tremolite, that can be found in certain types of rocks that are mined to extract chrysotile, has been indicated as the real cause of chrysotile-induce mesothelioma (11).

However, the current scientific evidence suggests that the clearance of chrysotile from lungs is much quicker compared to amphiboles, and this is the main explanation of the lower carcinogenic potency of chrysotile in humans (1, 4, 12).

Indeed, an extensive review published in 2000, based on epidemiological data, concluded that the chrysotile, amosite, and crocidolite contributed to the specific risk of mesothelioma, respectively, in the ratio 1:100:500 (1). The same paper stated a less marked difference between the three commercial types of asbestos in relation to the risk of lung cancer, with a ratio chrysotile/amosite of 1:10 and chrysotile/crocidolite of 1:50. Later, other authors recalculated these estimated considering the clearance of amphibole and found potency ratios for, respectively, crocidolite, amosite, and chrysotile of 26:14:1 (13).

A more recent review, based on *in-vitro* studies on biodurability of different kinds of asbestos fibers, as well as on animal models, human lung burden studies and epidemiological data, concludes that low exposures to chrysotile do not present a detectable risk to health, due to its rapid clearance, underlying the lower carcinogenic potential. Moreover, the authors suggest that even high exposures, if of short duration, bring about a low risk for health (14). However, the chrysotile rapid clearance, generally recognized and accepted, was questioned by Feder et al. who performed the only longitudinal study on fiber lung content, comparing biopsies, broncho-alveolar lavage, and postmortem data of the same 12 patients (15). They demonstrated that chrysotile fibers can be detected after several years after the cessation of exposure. However, the exact amount of chrysotile and amphiboles to which the subjects were exposed during life was not known, and therefore it is not possible to quantify how much chrysotile have been already cleared from the lungs before the first observation.

The aim of the present study was to evaluate the amount, the dimensional characteristics and the presence of asbestos in lungs (measured using SEM-EDS) in a series of deceased subjects who were certainly exposed to asbestos during their life. Moreover, we investigated possible correlations between the concentration of asbestos fibers (in general and considering each asbestos type), as well as their dimension (length, width and l/w ratio) and the duration of exposure, the latency (in case of MM), the survival time since the diagnosis of MM and the time elapsed between the end of exposure and death.

The subjects of this study used to live in Broni, a small town in Pavia province (Northern Italy), or work at the asbestos-cement plant located there. The production of asbestos-cement plant in Broni started in 1932. The standard asbestos cement blend was made up of Portland cement 325, chrysotile and crocidolite, while amosite were used only as an additive (16). Crocidolite use was stopped in 1987. Amosite was used until 1990, while chrysotile until 1993 (16). Until the 1960s, about 8,000 tons per year of asbestos cement products were produced, than the manufacturing increased to 100,000 tons/year (16). Until the 1970s, the manufacturing of tubes, sheets, chimneys, pipes, and other special products in asbestos cement was carried out in seven different production lines, where all mixing procedures were manual (16). Then, the production processes were improved and, since the early 1980s, a close-circuit mixer to prepare all the asbestos cement blends had been introduced (16). In the same years, ventilation units were installed to aspirate and filter the dust. Before 1981, environmental monitoring was never performed. Between 1981 and 1990, air quality was evaluated several times and no value exceeding the threshold (2 fibers/cc) was detected in between 1981 and 1984 (16). In 1990, 7 out of 44 samples exceeded 0.4 fibers/cc. In 1988 a breakdown of the sack shredder had been reported, and, as a consequence, concentrations over 10,000 fibers/cc and 2000 fibers/cc were measured for chrysotile and amosite, respectively (16). In 1993, the production was permanently stopped due to the national ban on the extraction, import, export and use of asbestos (Italian Law 257/1992). From the records of the trial we know that asbestos used in Broni came from asbestos mines located in Russia (chrysotile) and South Africa (crocidolite and amosite).

## Materials and Methods

### Population and Study Design

One hundred eighty-eight subjects died with ARD (MM and asbestosis), all of them living in Broni or occupationally exposed to asbestos, were the eligible people. A sample of 78 subjects was selected from the entire group of eligible using a non-proportional stratified random sampling by type of asbestos exposure, 26 for anthropogenic environmental exposure, 26 for occupational exposure, and 26 for both types of exposure. For the present study 72 cases were evaluated: in the group with occupational exposure 6 cases were excluded due to technical issues (problems in the chemical digestion leading to unsuitable samples for SEM-EDS examination). All of those with anthropogenic environmental exposure used to live nearby the asbestos cement plant and six of them had also household exposure.

A retrospective study design was applied. The study protocol was approved by the reference ethical committee for University of Pavia.

Data and information used in the present work derived from the archives of Forensic Medicine Department of the University of Pavia from 2000 to 2018. For each subject, during the autopsy whole lungs were collected, formalin-fixed, and stored for further examination. All of them had a diagnosis of MM or asbestosis, confirmed, in each case, by a forensic autopsy followed by histological examination. During the autopsy, the whole lungs had been collected, formalin-fixed and stored.

Regarding the type of exposure, in this paper the term “anthropogenic environmental exposure” is adopted, referring to people who lived in an area with air dispersed asbestos from the asbestos-cement plant (17, 18). The term “occupational exposure” refers to people who worked in contact with asbestos (19), even if, in some cases, not on a daily basis.

### SEM-EDS Investigation

For each subject, a sample of 0.25 g of formalin fixed lung tissue (inferior lobe) was prepared and inorganic fibers were investigated using SEM-EDS (JEOL JSM IT300LV-EDS Oxford INCA Energy 200 with INCA X-act SDD detector) according to the protocol described by Belluso et al. (20). According to respirable fiber definition (21), only those with, length > 5 μm, width < 3 μm length-to-width ratio > 3:1 (including asbestos-chrysotile, crocidolite, amosite, tremolite asbestos, actinolite asbestos, and anthophyllite asbestos) have been considered. The minimum width of fibers detectable by SEM-EDS is 0.2 μm, due to the working conditions and the technique (resolution limits of the instrument, type of sample preparation, instrumental parameters). Since the technique here used does not allow univocal identification of certain minerals having similar chemical composition and analogous morphology, it is not possible to distinguish chrysotile from asbestiform antigorite and tremolite asbestos from actinolite asbestos. Therefore, we used, respectively, the following mineral group names: chrysotile/asbestiform antigorite and tremolite/actinolite asbestos.

The number of total detected asbestos fibers were normalized to 1 gram of dry tissue as indicated by international guidelines (22, 23) reporting the concentration in terms of burden of asbestos per gram of dry lung tissue weight: ff/gdw. The concentrations of asbestos fibers, as well as the concentrations of the various types of asbestos, were calculated.

### Statistical Analysis

Descriptive analysis was carried out using means and standard deviation for the quantitative variables or median and 25th and 75th (IQR or Interquartile range) if the normality was not respected, and percentages for the qualitative ones. The Shapiro-Wilk's test was applied to assess normality. To evaluate the differences in quantitative variables among the exposure groups and the histological type of mesothelioma the analogous non-parametric test of analysis of variance (Kruskal-Wallis's test) was applied. If these analyses were significant the appropriate *post-hoc* test was used. The non-parametric unpaired *t* test (Mann-Whitney test) was performed to investigate differences in quantitative variables between subjects died for mesothelioma or for other causes. The relationships among the dimensional characteristics of the asbestos fibers (length and length/width ratio) and duration of exposure, time elapsed since the end of exposure, latency were tested using Spearman's rank correlation (rho).

A *p* < 0.05 was fixed as significant apart from the *post hoc* test, when the Bonferroni's correction for multiple test was used. All analyses were performed using STATA software v15.1 (StataCorp, College Station, USA).

## Results

In 62.5% of the samples the amount of detected asbestos was well-below the threshold considered demonstrative for occupational exposure, that is 0.1 × 10^6^ of amphibole asbestos longer than 5 μm per 1 gdw or more than 10 × 10^5^ amphibole asbestos longer than 1 μm per 1 gdw as measured by electron microscopy in a qualified laboratory (24). Among the 45 subjects above mentioned, 24 lived nearby the industry (in 9 of them no asbestos at all was detected); one of them was a safety inspector, who used to spend about 1 week per year at the plant; 12 of them were employed at the asbestos-cement plant as workmen; one of them was a surveyor who worked at the plant for 5 years; 2 worked at the railway station located inside the plant; the remaining five worked in other industries where asbestos was used in several ways (insulation, filtration, asbestos-cement artifacts production).

In 21 subjects (29.1% of cases), no asbestos was found, despite the anamnesis documented certain exposure. Of these, 8 had occupational exposure.

In regard to the kinds of asbestos, the most important aspect is the nearly complete absence of chrysotile/asbestiform antigorite. This kind of asbestos was detected (in extremely low quantity) in only one of the examined samples. Almost all the detected asbestos was classified as crocidolite or amosite. Very low quantities of tremolite/actinolite asbestos and anthophyllite asbestos have also been found. In detail, crocidolite was the most abundant kind of asbestos (51% of the total amount of asbestos), followed by amosite (46%), tremolite/actinolite asbestos (3.3%), and anthophyllite asbestos (0.9%).

The mean length and width of asbestos fibers were calculated in each case: they ranged, respectively, between 6 and 58 μm, and between 0.5 and 2 μm. The median length, width and length/width ratio of asbestos fibers was not significantly different among three types of exposure (Table 1). However, the highest length (23.8 μm) and length/width ratio (47.8 μm) was observed in occupationally exposed subjects.

**Table 1:** Dimensional characteristics of asbestos according to the kind of exposure.

	**Both Exposures**	**Anthropogenic environmental exposure**	**Occupational exposure**	Test^*^ and *p*-value
	***N* = 26**	***N* = 26**	***N* = 20**	
**Mean length of asbestos fibers (μm)**				
Median (IQR)	20.6 (13.9–26.2)	20.3 (18.1–31.4)	23.8 (15.8–40.3)	KW = 0.53 0.764
**Length/width ratio**				
Median (IQR)	32.9 (24.8–43.7)	29.0 (21.0–51.3)	47.8 (18.0–66.3)	KW = 0.40 0.818

* *Kruskall Wallis test = KW*.

Moreover, no relevant difference was found in mean length (nor in length/width ratio) of asbestos between subjects who died of MM and those who died from other asbestosis-related causes (Table 2).

**Table 2:** Dimensional characteristics of asbestos according to the cause of death.

	**Asbestosis**	**Malignant mesothelioma**	Test^*^ and *p*-value
	***N* = 13**	***N* = 59**	
**Mean length of asbestos fibers (μm)**			
Median (IQR)	24.2 (19.7–36.7)	19.3 (15.1–32.2)	MW = 1.40 0.159
**Length/width ratio**			
Median (IQR)	43.7 (30.2–50.4)	29.0 (20.3–58.0)	MW = 1.70 0.087

* *Mann-Whitney test = MW*.

For all the 72 subjects, a possible correlation between the amount of asbestos fibers (as well as of each kind of asbestos) and, respectively, the duration of exposure, the latency and the survival time since diagnosis (in cases with MM), the time elapsed since the end of exposure, was investigated.

Moreover, the correlation between the dimensional characteristics of the asbestos fibers (mean length and length/width ratio) and the same variables was examined.

For the assessment of correlation between asbestos amount and dimensions and the mentioned time intervals, both environmental exposure and occupational exposure were considered, as 26 subjects had both kind of exposures.

The duration of occupational exposure to asbestos ranged from 6 to 480 months (median = 264, IQR 108–360 months), whereas anthropogenic environmental exposure was found to last between 36 and 720 months (median= 414, IQR 258–576). Unexpectedly, the amount of asbestos fibers, as well as the amount of the single species of asbestos, did not show any significant correlation with the duration of exposure, regardless the type of exposure considered, occupational or anthropogenic environmental (Supplementary Table 1).

The latency (calculated only in MM cases), defined as the time elapsed between the beginning of exposure and the diagnosis, ranged from 16 to 60 years considering occupational exposure (median = 41 years, IQR 33–48) and 19–80 years (median = 53 years, IQR 42–65) considering environmental exposure.

No correlation was found between the amount of asbestos and the latency period (the time between the first exposure and the diagnosis of MM) (Supplementary Table 1).

The survival time since the diagnosis of MM ranged between 1 and 379 months (median = 15 months, IQR 9.5–28.5) and was not related to the amount of asbestos in lungs (Supplementary Table 1).

The time elapsed between the end of exposure and death ranged between 8 and 44 years (median = 21 years, IQR 18–26). The amount of asbestos did not show any correlation with the time since the end of exposure (Supplementary Table 1).

**Figure 1:**
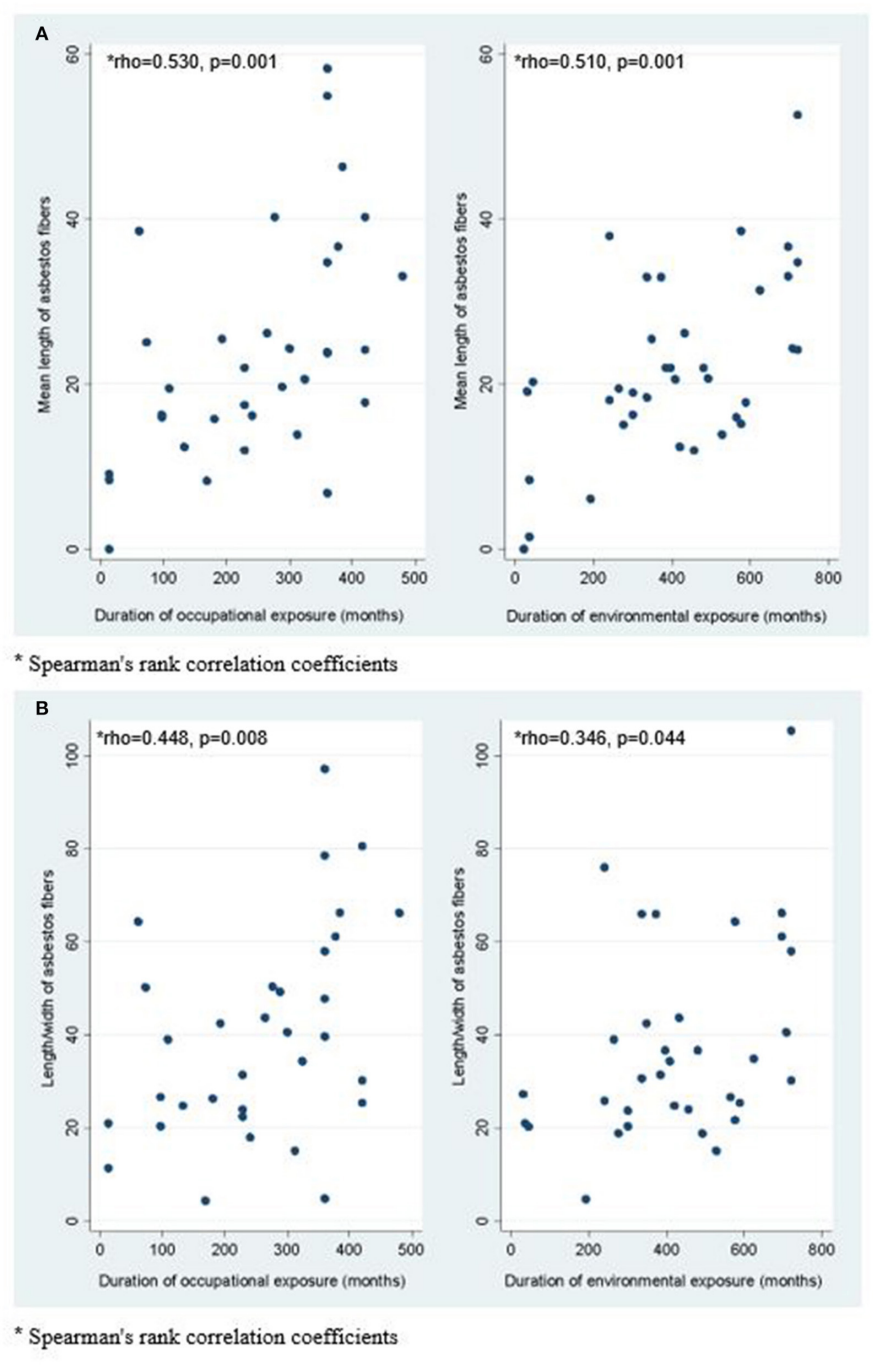
Correlation between duration of occupational and environmental exposure and mean length of asbestos fibers **(A)**; Correlation between duration of occupational and environmental exposure and length/width ratio of asbestos fibers **(B)**.

The mean length of asbestos fibers and the length to width ratio were positively related to the duration of exposure to asbestos (Figure 1). No other statistically significant correlations were found for the other chronological variables considered (Table 3).

**Table 3:** Spearman's rank correlation coefficients of correlation between mean length, length/width ratio, and the chronologic variables considered.

	**Period of occupational latency (years)**		**Period of environmental latency (years)**		**Survival time since diagnosis of MM (months)**		**Time since the end of exposure (years)**	
	^*^rho	***p*.value**	**rho**	***p*.value**	**rho**	***p*.value**	**rho**	***p*.value**
Mean length of asbestos fibers (μm)	−0.050	0.795	0.248	0.155	0.095	0.514	−0.176	0.210
Length/width ratio	−0.233	0.232	0.041	0.820	0.227	0.124	−0.157	0.276

* *rho, Spearman's rank correlation coefficients*.

## Discussion

The asbestos fiber burden in lungs is generally determined in autoptic samples, using analytical electron microscopy, mostly after several years since the cessation of exposure. Therefore, even though this kind of analysis provides relevant information about asbestos exposure during life, it does not reflect the exact entity and quality of past exposure. In fact, asbestos clearance from lungs that occurred before the subject's death plays a crucial role in determining the lung content that can be measured after death. Thus, the results of SEM-EDS analysis must be interpreted carefully, being aware of when the exposure had ceased. More importantly, the bio-persistence of the various kinds of asbestos is an essential characteristic which influences greatly the health hazardousness of asbestos.

Our results demonstrated the occurrence of asbestos clearance (regarding, in particular, chrysotile) from human lungs, based on the following findings.

First, it must be highlighted 62.5% of the investigated samples, much less asbestos than expected was found (in particular, the concentration of asbestos in lungs was well-below the threshold considered significant for occupational exposure). Moreover, in 29.2% of them no asbestos was detected. Those findings are certainly unexpected, given that, even though the intensity of exposure is very different from subject to subject (and not exactly verifiable), all of them had a documented exposure to asbestos during life, either occupational or anthropogenic environmental, or both of them in about one third of the cases.

Secondly, chrysotile was found in only one subject of the 72 samples.

In all the 72 subjects of this study, the asbestos exposure had ceased at least 8 years before death (median = 21 years). Thus, the finding of much less asbestos than expected is likely to be related to its previous removal from the lung microenvironment. In fact, asbestos lung clearance is known to involve mainly chrysotile, that represents the less bio-persistent asbestos type (9, 14, 25).

Therefore, the possible explanation for the low amount or even the absence of asbestos, despite the certain exposure during life, may be that in those subjects the asbestos content of lungs consisted mainly of chrysotile, which had been cleared from the lungs after the end of exposure. This hypothesis is consistent with the data about the production at the asbestos-cement factory located in Broni, according to which chrysotile, together with crocidolite, was the most utilized kind of asbestos (16).

The actual biopersistence of chrysotile is still the subject of an open debate. The chemical composition and structure of chrysotile, which is different from amphiboles, explains its lower biodurability (4, 14). In fact, the acid microenvironment dissociates the magnesium from the structure of chrysotile, making the fiber easily breakable (14). After fragmentation, the small fibrils are engulfed by macrophages (26) and removed from lungs.

Most studies about asbestos clearance were performed on animals. Such experiments showed that, after 90 days since the end of exposure to chrysotile, very few fibers remained detectable, compared to amphiboles after the same period of time (27, 28). Published data about chrysotile clearance, both in humans and animals, are contradictory. For instance, a work on humans pointed out the presence of chrysotile as late as 60 years after exposure (29). A recent longitudinal study on a small number of human lung samples showed that chrysotile can persist for as long as 37 years (15), and it is observable in human lung samples using a high-resolution electron microscope, a FEG-SEM.

On the contrary, the present study suggests a complete pulmonary removal of chrysotile. This finding has great implications from an occupational health point of view, as chrysotile is the prevalent kind of asbestos that is still mined and used in many countries. Moreover, this hypothesis may have a great importance in forensic investigations and in processual debates: indeed, if the amount of detected chrysotile is lower than the threshold considered indicative for occupational exposure, this can be due to clearance.

Lung clearance does not regard only chrysotile, but was experimentally demonstrated on animal models for amphiboles by Rendall and Du Toit, who estimated a half-life of about 50 months for crocidolite and 18 months for amosite (28). These results correlate well with observations conducted on humans by the same author (30). The predominance of crocidolite and amosite in our samples in lungs of almost all the subjects is consistent with the data about the plant (16, 31) and reflects a strong biopersistence of amphiboles in lungs.

Unlike Du Toit, we did not find any relationship between fiber burden and time since last exposure (32) (Supplementary Table 1). Other authors found a positive correlation between the asbestos concentration in lungs and the duration and intensity of exposure, as well as a negative correlation between asbestos amount and the time since the end of exposure (33). De Klerk et al. performed TEM lung content analysis on lung samples of Australian crocidolite workers (Wittenoom industry, Gorge) and compared the results with airborne concentration of asbestos (33). They found a significant association between asbestos amounts in lungs and both duration and intensity of exposure, as well as a negative correlation between asbestos in lungs and time since last exposure. Probably, the exposure to asbestos of the subjects here analyzed (considering especially amphiboles, that are not subjected to degradation as quickly as chrysotile) was sufficiently high to exceed the clearance rate of amphiboles, resulting in a fiber overload and in an impaired macrophages function. This explains the absence of a negative correlation between lung amphibole burden and time elapsed since last exposure (34). Moreover, when high amounts of asbestos are inhaled, some of them end up in the interstitium (as can be commonly observed in asbestosis cases): here they are “sequestered” from clearance and are inevitably retained, due to fibrosis and scarce vascularization. Anyway, overload of phagocytosis is not even necessary to impair lung clearance when the particles are highly cytotoxic, like amphiboles. Indeed, asbestos, and especially amphiboles, can escape phagocytosis and clearance also at lower doses, because they alter the chemotactic stimuli and slow the cell extravasation and random migration (34).

Churg and De Paoli, in 1988 (7), hypothesized that inhaled chrysotile might end up as two populations: one is cleared quickly from the air spaces and another, composed of fibers which persist after reaching the interstitium. Other authors suggests that chrysotile clearance from the “short term” compartment is extremely rapid, whereas its removal from the “lung term” sequestration compartment is slow (25). Our findings suggest that chrysotile, as opposite to amphiboles, is completely removed even from the “long term” sequestration after <8 years, that is the shortest period since the end of exposure in the subjects of this study.

We have to consider also another possible explanation. There is good evidence that *in-vivo* longitudinal splitting of amphibole fibers occurs in the lungs of asbestos workers, as documented in a series of studies by Germine and Puffer (35–37). As fibers are cleared or dissolved they are replaced, or the number of fibers is balanced, by the new fibers split off of existing fibers. In addition, *in-vivo* longitudinal splitting of amphibole asbestos fibers has been documented in a 2-year study of intrapleural injection and intratracheal instillation of asbestos in rats. *In-vivo* splitting during 2 years produced four times greater the number of fibers than the number originally instilled (38). This mechanism can explain why the concentration of asbestos in lungs dies not decrease with time.

Moreover, our findings did not show any significant correlation between duration of exposure and asbestos fibers concentration (gdw) observed by SEM-EDS in lung samples (Supplementary Table 1). This finding, in contrast with epidemiological studies that assume that a longer duration of exposure reflects a heavier fiber burden in lungs (39), may reflect, again, the quick degradation of chrysotile, which explains the absence of this logical correlation.

The survival time of MM patients is influenced by a lot of factors, for instance age, type of neoplasm, socio-economic status. According to our data, the lung fiber burden does not seem to affect the survival time since diagnosis (Supplementary Table 1). It means that the concentration of asbestos (not cleared from lungs) does not influence the aggressiveness nor the progress of the disease. On the contrary, the absence of asbestos exposure (based on anamnestic assessment) has been previously related to a longer survival, compared to occupational exposure (40). Moreover, a recent paper conducted on a large number of MM patients supported hypothesis that subjects with demonstrated asbestos exposure have shorter survival times than non-exposed cases (41). Our findings, based on lung content analysis, seem to confute such studies (that were based only on clinical and anamnestic data).

Regarding the dimensional characteristics of asbestos, for this study we considered only fibers longer than 5 μm, width <3 μm, and length/width ratio > 3:1, according to the widely accepted definition of fiber (21). We observed an interesting correlation (Figure 1): the length of asbestos fibers, as well as the length/width ratio, increases with the duration of exposure, but does not correlate with the time which elapsed since the end of exposure. This finding might be due to the longitudinal splitting of amphibole fibers, reported by Germine and Puffer and by Cook et al. (35–38), that explains both the higher concentration of long fibers and the increasing of the length/width ratio of the amphibolic fibers with time. The longitudinal splitting that occurs with time must be taken into account when measuring the lung burden of asbestos in subjects whose exposure had ceased before death.

## Conclusions

The present findings, obtained by asbestos lung content analysis using SEM-EDS in a significant number of exposed individuals, allowed to point out that chrysotile can be completely removed from human lungs after about 8 years since the end of exposure. Moreover, this study suggested a long biopersistence of amphiboles (crocidolite, amosite, tremolite/actinolite asbestos, and anthophyllite asbestos). This is relevant from a public health point of view, as biodurability has been suggested to be an important determinant of health hazardousness of asbestos (42). We have to point out a limitation of the study: according to the respirable fiber definition (21), here we chose to consider only fibers with length > 5 μm, width <3 μm length/width ratio > 3:1. Moreover, due to the instrumental working conditions, the minimum width of the detected fibers is 0.2 μm. Such limitations led to undetect fibers thinner than 0.2 μm, whose pathogenicity cannot be ruled out (43).

Another limitation of the present study is that only lung fiber burden, and not in pleural tissue, is measured. In fact, the problem of correlation between the concentration and the kind of asbestos in lungs and pleural tissue has been addressed in literature, finding that fibers in pleural space may be different (both quantitatively and qualitatively) from those in lung (44–46). However, we are convinced that studying asbestos in lungs is important in order to understand more about deposition and clearance mechanisms, as after inhalation, in order to reach pleura, asbestos must pass through the lung parenchyma. Asbestos concentration in pleural tissue will be addressed by further studies in the present series.

The present results suggest, as well, that the finding of no asbestos in lungs using SEM-EDS [considered currently the gold standard (24)] cannot rule out the attribution of MM to asbestos (in particular, chrysotile) inhaled in an occupational setting. This point is of crucial importance from a legal point of view. What pointed out above suggests, as well, that the cut off considered demonstrative for occupational exposure should be re-evaluated.

All of the individuals in our series with no detected asbestos in their lungs died of MM. This might mean that the carcinogenic effect of asbestos is not eliminated by its clearance in lungs. As already stated by Toyokuni (47), maybe the carcinogenic effect on mesothelium is exerted once the asbestos is removed from the lungs and transmigrates in the pleural space through the lymphatic system. This may explain the long latency period required for the onset of MM. On the other hand, the duration of exposure, as well as the time elapsed since the end of exposure to death, do not correlate with the amount of fibers in lungs, suggesting that the deposition and clearance patterns of both amphiboles and chrysotile are complex and they have to be studied more deeply.

## Data Availability Statement

The raw data supporting the conclusions of this article will be made available by the authors, without undue reservation.

## Ethics Statement

The studies involving human participants were reviewed and approved by the reference ethical committee for University of Pavia on 4th July 2018 (Protocol No. 2018060636). Written informed consent for participation was not required for this study in accordance with the national legislation and the institutional requirements.

## Author Contributions

SV, CP, and CC designed the study. SC, SB, and EC collected and organized the data. PB and SV performed the statistical analysis and designed tables and images. CC, CP, and EB supervised the work. SV wrote the original draft. SC, EB, and CC reviewed and edited the draft. All authors have read and agreed to the published version of the manuscript.

## Conflict of Interest

CC acted as experts for the court, the public prosecutors and the defense in asbestos-related litigations NOT related to the subjects of this paper. The remaining authors declare that the research was conducted in the absence of any commercial or financial relationships that could be construed as a potential conflict of interest.
