# Supplementary material for: Evaluation of Deposition and Clearance of Asbestos (Detected by SEM-EDS) in Lungs of Deceased Subjects Environmentally and/or Occupationally Exposed in Broni (Pavia, Northern Italy)
Source: Front Public Health. 2021 Jul 20;9:678040. doi: 10.3389/fpubh.2021.678040 (PMC8329042; doi:10.3389/fpubh.2021.678040)
Supplement: Supplementary file 1 [file Table_1.DOCX]

|  | Duration of occupational exposure (months) | | Duration of environmental  exposure (months) | | Period of work latency (years) | | Period of environmental latency (years) | | Survival time  (months) | | Time since end of  Exposure (years) | |
| --- | --- | --- | --- | --- | --- | --- | --- | --- | --- | --- | --- | --- |
|  | **rho** | **p.value** | **rho** | **p.value** | **rho** | **p.value** | **rho** | **p.value** | **rho** | **p.value** | **rho** | **p.value** |
| Asbestos fibers per gram of dry weight lung tissue | 0.286 | 0.063 | 0.180 | 0.201 | -0.111 | 0.511 | 0.023 | 0.870 | 0.092 | 0.453 | -0.062 | 0.602 |
| Chrysotile/asbestiform antigorite fibers | 0.012 | 0.936 | - | - | -0.140 | 0.406 | - | - | 0.177 | 0.147 | 0.096 | 0.423 |
| Crocidolite fibers | 0.274 | 0.074 | 0.222 | 0.112 | -0.080 | 0.634 | 0.003 | 0.979 | 0.082 | 0.505 | -0.119 | 0.319 |
| Amosite fibers | 0.263 | 0.087 | 0.070 | 0.620 | -0.114 | 0.499 | 0.036 | 0.799 | 0.103 | 0.403 | 0.095 | 0.426 |
| Anthophyllite fibers | -0.077 | 0.620 | -0.146 | 0.300 | -0.350 | 0.137 | -0.038 | 0.788 | -0.150 | 0.220 | 0.045 | 0.705 |
| Tremolite fibers | 0.178 | 0.253 | 0.062 | 0.654 | 0.010 | 0.949 | 0.142 | 0.325 | 0.066 | 0.589 | -0.210 | 0.077 |

SUPPLEMENTARY TABLE 1: correlations between amount of asbestos and of each asbestos type and the duration of exposure (respectively, occupational and environmental), latency (respectively, occupational and environmental), survival, time since the end of exposure.
